# miRNAs in Newt Lens Regeneration: Specific Control of Proliferation and Evidence for miRNA Networking

**DOI:** 10.1371/journal.pone.0012058

**Published:** 2010-08-11

**Authors:** Kenta Nakamura, Nobuyasu Maki, Albert Trinh, Heidi W. Trask, Jiang Gui, Craig R. Tomlinson, Panagiotis A. Tsonis

**Affiliations:** 1 Department of Biology and Center for Tissue Regeneration and Engineering, University of Dayton, Dayton, Ohio, United States of America; 2 Departments of Medicine and Pharmacology and Toxicology, Dartmouth Hitchcock Medical Center, Norris Cotton Cancer Center, Dartmouth College, Lebanon, New Hampshire, United States of America; Midwestern University, United States of America

## Abstract

**Background:**

Lens regeneration in adult newts occurs via transdifferentiation of the pigment epithelial cells (PECs) of the dorsal iris. The same source of cells from the ventral iris is not able to undergo this process. In an attempt to understand this restriction we have studied in the past expression patterns of miRNAs. Among several miRNAs we have found that mir-148 shows an up-regulation in the ventral iris, while members of the let-7 family showed down-regulation in dorsal iris during dedifferentiation.

**Methodology/Principal Findings:**

We have performed gain- and loss-of–function experiments of mir-148 and let-7b in an attempt to delineate their function. We find that up-regulation of mir-148 caused significant decrease in the proliferation rates of ventral PECs only, while up-regulation of let-7b affected proliferation of both dorsal and ventral PECs. Neither miRNA was able to affect lens morphogenesis or induction. To further understand how this effect of miRNA up-regulation is mediated we examined global expression of miRNAs after up-regulation of mir148 and let-7b. Interestingly, we identified a novel level of mirRNA regulation, which might indicate that miRNAs are regulated as a network.

**Conclusion/Significance:**

The major conclusion is that different miRNAs can control proliferation in the dorsal or ventral iris possibly by a different mechanism. Of interest is that down-regulation of the let-7 family members has also been documented in other systems undergoing reprogramming, such as in stem cells or oocytes. This might indicate that reprogramming during newt regeneration shares common molecular signatures with reprogramming in stem or germ cells. On the other hand that miRNAs can regulate the levels of other miRNAs is a novel level of regulation, which might provide new insights on their function.

## Introduction

Lens regeneration in adult newt is one of the most interesting cases of organ regeneration. It also represents a clear case of transdifferentiation. Specifically, after lentectony the pigment epithelial cells (PECs) of the dorsal iris dedifferentiate and then differentiate to lens cells. The same cells from the ventral iris cannot undergo these events [1], [2]. Nevertheless, ventral iris PECs do re-enter the cell cycle and proliferate (but in lower levels) and also are quite active in expressing regulatory genes at the dorsal iris [3], [4], [5]. Thus, we have been entertaining the idea that the ventral iris might initiate some of the events of dedifferentiation but then a repression event stops it from further commitment that would lead to regeneration. In an attempt to understand this phenomenon and address the repression issue we have studied in the past expression of miRNAs in both dorsal and ventral iris. miRNAs (20–22 nt long) are know factors that suppress expression of many genes by binding to target sequences in the 3′UTR of many mRNAs. We identified several miRNAs that were differentially regulated in the dorsal and ventral iris [6]. Most notably we found that mir-148 was upregulated in the ventral iris. Likewise members of the let-7 family were found to be down-regulated during the process of dedifferentiation in dorsal iris. In the present study we undertook loss-and gain-of function experiments for mir-148 and let-7b to delineate their function during the process of regeneration. We found that up-regulation of mir-148 most likely controls specifically the rate of proliferation in ventral PECs, while let-7b control proliferation of both dorsal and ventral PECs. Neither miRNA affected lens morphogenesis. This is consistent with the expression patterns of mir148 and indicates that control of proliferation in dorsal and ventral iris might have different regulators. We then examined global miRNA expression due to up-regulation of mir-148 and let-7b. Interestingly we found a new level of regulation: perturbations in the expression of miRNAs can affect the expression of other miRNAs, indicating that miRNAs might act as in networks.

## Methods

### Iris pigment epithelial cell culture

Usage of animals has been approved by the Institutional Animal Care and Use Committee (IACUC) of University of Dayton (Assurance Number A 3092-01).

Newts were anesthetized with 0.1% ethyl 3-aminobanzoate methanesulfonic acid salt, and animals were sacrificed immediately. Eyes were cut in half and iris was collected in Hank's solution, then remaining retinal tissues and marginal place of iris were totally removed from them. Iris tissues were divided dorsal and ventral parts and placed in culture medium (65% diluted L-15 with 10% FBS, containing 100 µg/mL kanamycin sulfate and 2.5 µg/mL Amphotericin B solution). Irises were treated with dispase (GIBCO, ∼5 mg/mL) for 2–3 hrs, followed by removal of the stroma. Collected iris tissue/cell layer was treated by 2.5 mg/mL trypsin for 2 hrs, and then isolated cells were placed on collagen I-coated plates. Cells were kept in culture for at least one week before used.

### Transfection of cultured PECs

For transfection, Lipofection by Lipofectamine (invtrogen) with plus reagent was performed. For down-regulation of micro RNA, miRCURY™ LNA knockdown probes (EXIQON) were used, and for up-regulation, Ambion Pre-miR™ miRNA Precursor Molecule was used. For controls we used the following: Ambion's Pre-miR negative control #1 in the up-regulation experiments and miRCURY™ knockdown scramble-miR for down-regulation. The final concentration was 1.6 µM.

### Cell aggregation and implantation

After transfection, cells were kept for 4 days, then treated by 1.5 mg/mL dispase for overnight. Cells were collected to 1.5 mL tubes and washed with medium, then divided in aliquots containing 3000–5000 cells each. After they aggregated well (2–3 days), each aggregate was implanted into lentectomized eyes. Animals were kept for 1 month to induce regeneration before they eyes were examined for effects on the regeneration process. This method can be used for transient expression of transfected genes for induction of regeneration (3).

### Immunohistochemistry

For immunohistochemistry, eyeballs were fixed in 4% Paraformaldehyde in PBS at 4°C, overnight. Then, tissues were dehydrated and embedded in paraffin. 15 µm sections were mounted on gelatin-coated slides. For a primary antibody, mouse monoclonal anti-crystallin [7] was used at 4°C overnight. For detection 1∶100 Alexa 488 conjugated anti-mouse IgG (invitrogen).

For BrdU incorporation, 5 days after transfection, cells were treated with 0.25 mg/mL BrdU for 24 hrs, then washed with PBS and fixed with 3∶1 mixture of Methanol: Acetic Acid 10 min. After washing with 100% MetOH, they were dried-up and kept in −20°C until used. At that time cells were washed with 2x SSC, treated with 0.5% saponin/0.5% Triton X-100 in 2x SSC, 1 hr, followed by 1M HCl for 5 min. They were then immediately washed with 2xSSC three times before the buffer was replaced to TN-Buffer (0.1 M Tris-HCl pH 7.5+150 mM NaCl) for 10 min, then blocked by TNB (TN-Buffer with 0.5% Blocking reagent) (Perkin Elmer, FP1020) at room temperature. As primary antibody, CHEMICON MAB3510, mouse anti-BrdU, 1∶200 in TNB was used at 4°C for overnight. Cells were washed with TNT (TN-Buffer with 0.05% Tween 20), then treated with secondary antibody, 1∶100 Alexa 488 conjugated anti-mouse IgG (invitrogen) in TNT, 90 min at room temperature. After washing with TNT, wells were mounted by Vectashield with DAPI (Vector Laboratories) and then observed. Cells were counted for BrdU immunoreactivity and total cell number was counted by DAPI-stained nuclei. The BrdU/DAPI ratio was calculated as percentage. On average 5 samples were used. For statistics we used Student's t-test.

### miRNA microarrays

Lens tissue was homogenized in TRIzol Reagent (Invitrogen Corp., Carlsbad, CA) from which total RNA was isolated following the accompanying instructions [8]. Total RNA purity, quantity, and quality were determined using a NanoDrop spectrophotometer ND-1000 (Thermo Scientific, Waltham, MA) and Agilent Bioanalyzer 2100 (Agilent Technologies, Santa Clara, CA). miRNA was isolated from the total RNA preparation using the Flash Page™ gel system (Ambion, Austin, TX). The purified miRNA was amplified using the NCode™ miRNA Amplification System (Invitrogen Corp., Carlsbad, CA) from which the sense strand RNA was isolated using the PureLink™ Micro kit (Invitrogen Corp., Carlsbad, CA). The sense strand miRNA was labeled with cyanine-3 and used for hybridization on mouse miRNA microarray slides version 2 (Agilent Technologies, Santa Clara, CA). To our knowledge and experience miRNA microarray analysis, using arrays from other species is quite acceptable due to the high degree of sequence conservation between miRNAs (6). The data analysis was carried out by performing loess normalization [9] without background correction to pre-process the image files from the Agilent Feature Extraction software [10] and an intensity-based, modified t-test [11] to characterize the significance level of each feature using limma R packages from Bioconductor [12].

## Results and Discussion

### Tranfection and Luciferase Activity

**Figure 1:**
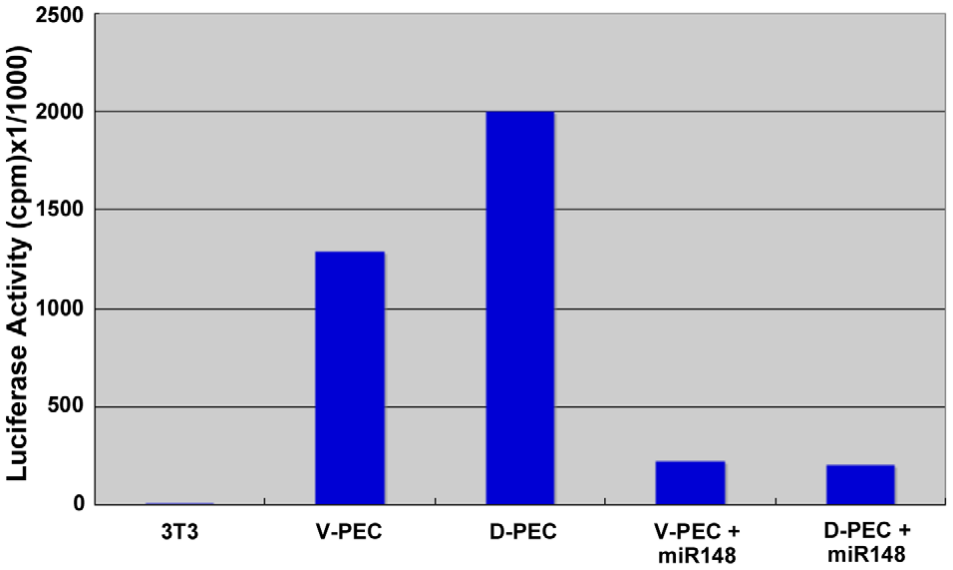
Luciferase activity. Luciferase activity in ventral and dorsal PECs transfected with a luciferase vector containing target target sequences for miR148. Note inhibition in cells that were co-transfected with miR-148. 3T3 cells were the untransfected control. The values are average of 20 measurements.

To ensure that transfections of miRNAs can specifically regulate mRNAs containing their target sequences, we tranfected PECs with a luciferase construct containing mir-148 target sequences. Luciferase activity was very high when compared with untransfected 3T3 cells (figure 1). Interestingly the activity in the ventral PECs was lower than the activity in the dorsal PECs. This is consistent with our previous finding that there is more mir-148 in the ventral iris than the dorsal. However, when the plasmid was co-transfected with mir-148 as well, luciferase activity was significantly decreased.

### Gain- and loss-of-function of miRNAs in dorsal and ventral iris

**Figure 2:**
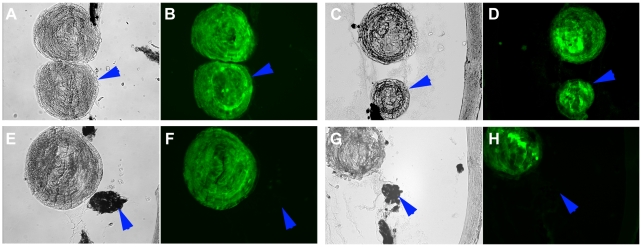
Gain of function. Effects of miR-148 and let-7b up-regulation on the capability of dorsal and ventral PECs to transdifferentiate to lens. Cells were transfected as described in methods and after they were aggregated they were implanted in lentectomized eyes. The eyes were examined 30 days later. A, B: dorsal PECs and E, F: ventral PECs, transfected with miR-148. C, D: dorsal PECs and G, H: ventral PECs transfected with let-7b. Note that only dorsal aggregates differentiated to lens (left panels are phase contrast and left panels stained with a crystallin antibody). Arrowheads indicate the induced lens from the dorsal aggregates or the un-induced ventral PECs aggregate.

**Figure 3:**
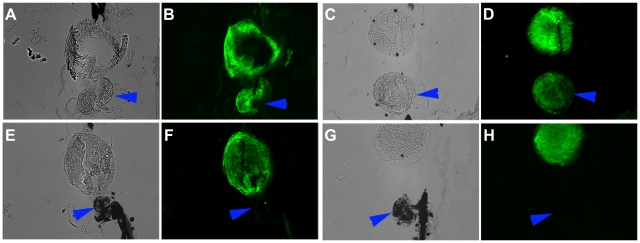
Loss of function. Effects of miR-148 and let-7b down-regulation on the capability of dorsal and ventral PECs to transdifferentiate to lens. Same description as in Figure 2 legend.

To delineate the function of mir-148 and let-7b during lens regeneration we performed gain- and loss-of-function experiments. For this dorsal or ventral PECs were transfected with pre-miR constructs (gain-of-function) or with antisense LNAs (loss-of-function). We followed the standard protocol to examine their effects on lens morphogenesis. After transfection, the cells remained in culture for two weeks. They were then aggregated and placed in a lentectomized eye. This transplantation procedure recapitulates the normal in vivo conditions. Only the dorsal aggregates transdifferentiate to lens, while the ventral ones do not [13]. Thus any effect on either dorsal or ventral iris can be assessed 30 days after transplantation. The overall results are shown in Table 1. Generally without any treatment, the transplantation protocol that we use will induce lens transdifferentiation in 75–100% of the dorsal aggregates, while there is never a case of ventral iris transdifferentiating to lens (0%). As can be seen from the results in none of the cases the ventral iris was induced to transdifferentiate. Also in all cases but the dorsal mir148 up-regulation, the percentage of transdifferentiation from the dorsal aggregates was within the normal range. Of interest is the fact that when dorsal cells were up-regulated with mir-148 there was only 50% of transdifferentiation. This seems somehow low, but we do not think is a significant effect. In Figure 2 we present cases of lens transdifferentiation from the dorsal aggregates with up-regulated mir-148 and let-7b and in Figure 3 cases of lens transdifferentiation from dorsal aggregates with down-regulated mir-148 and let-7b. In all induced cases the lens seems to be of normal morphology.

**Table 1:** Ratio of lens regeneration from gain or loss of function of miRNAs in PECs.

	**Untreated**	**miR-148a up**	**let-7b up**
Dorsal	9/12	4/8	8/9
Ventral	0/10	0/9	0/9
	Untreated	miR-148a down	let-7b down
Dorsal	9/12	3/3	3/4
Ventral	0/10	0/8	0/8

### Effects of mir148 and let-7b on PEC proliferation

**Figure 4:**
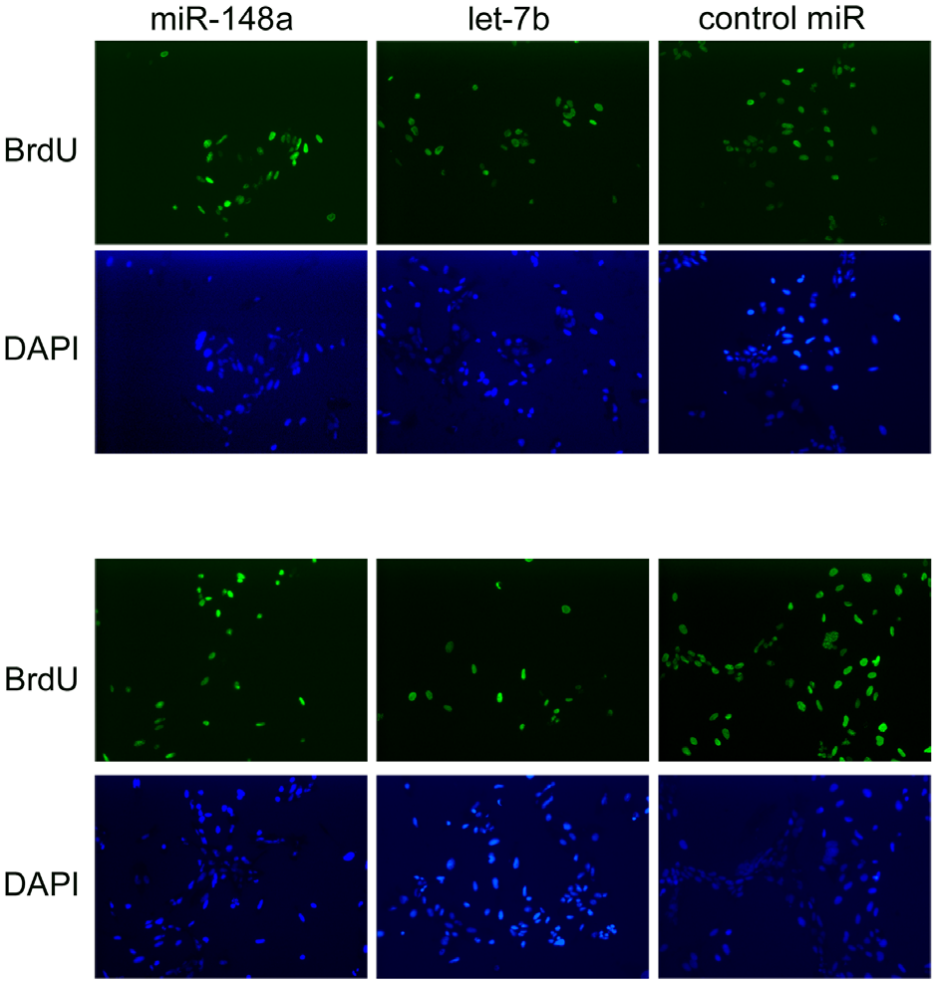
Proliferation experiments. Examples of proliferating cells transfected with miR-148 or let-7b. Top panels are dorsal PECs and low panels are ventral PECs.

**Figure 5:**
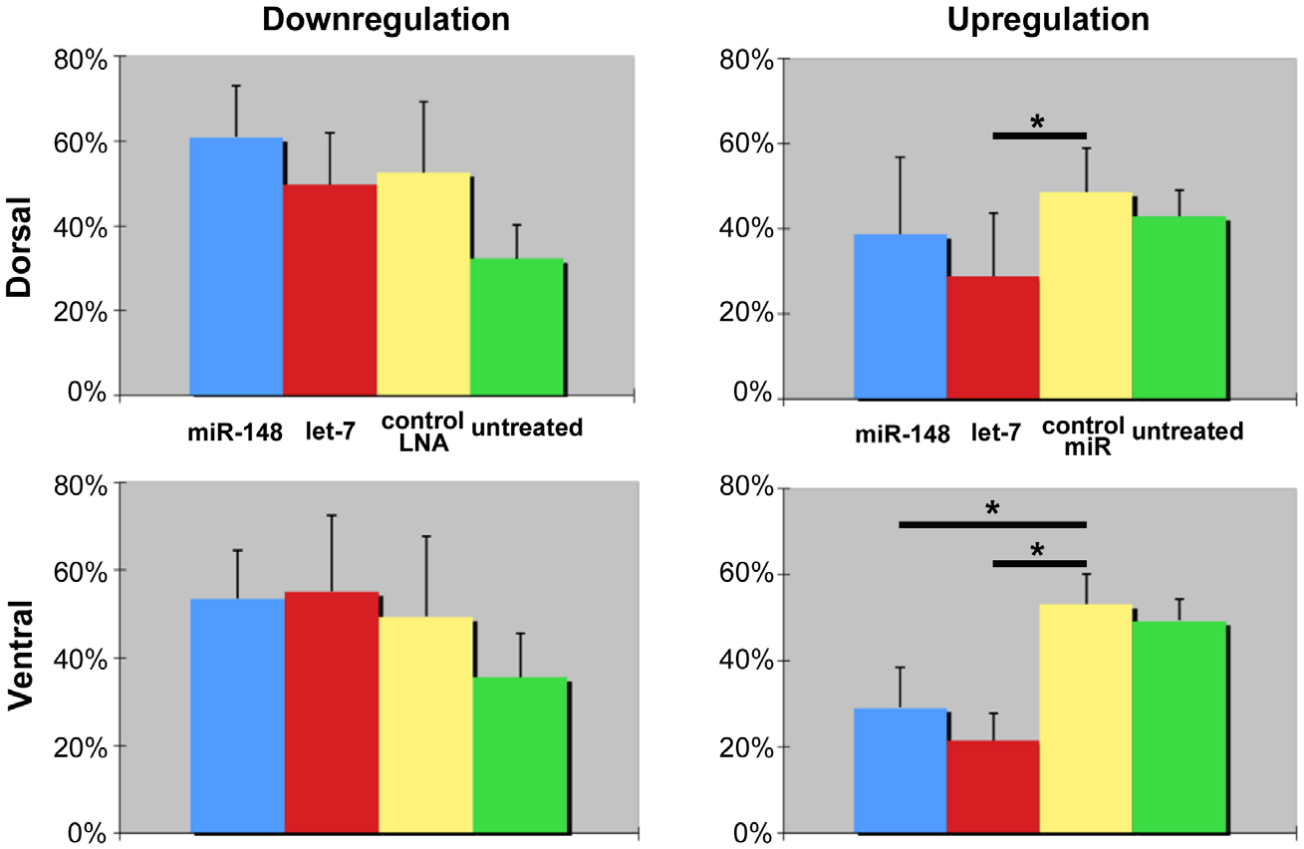
Quantitation of proliferation. Effects of gain or loss of function of miR-148 and let-7b on PEC proliferation.

Since both miRNAs were not able to induce the ventral PECs to transdifferentiate we hypothesized that maybe their effect is in controlling earlier events of regeneration. As mentioned in the introduction, the ventral iris initiates events of proliferation and gene expression. Since only the dorsal iris is able to proceed to transdifferentiate to lens there could be a differential effect on control of cell proliferation in dorsal and ventral iris. We, therefore, examined the effect on PEC proliferation after gain- and loss-of-function experiments. The results were quite interesting. We found that up-regulation of mir-148 had a significant inhibitory effect on ventral PECs only, while up-regulation of let-7b inhibited both ventral and dorsal PEC proliferation (figure 4,5). Interestingly, down-regulation of the two miRNAs did not affect cell proliferation. The interpretation of these results leads to the conclusion that proliferation of ventral iris PECs is regulated in a different way than in the dorsal iris PECs. mir-148 is ventral-specific. And we find that it affects proliferation only in ventral PECs. Therefore we believe that higher levels in the ventral iris must correlate with control of cell proliferation in these cells only. Down-regulation of mir-148 does not interfere with proliferation, but because the dorsal PECs were not affected with either mir-148 treatment it seems that they are indifferent to its presence. On the other hand, let-7b, which is down-regulated in the dorsal iris during dedifferentiation, does inhibit proliferation of the dorsal PECs, when up-regulated in these cells. In other words experimental up-regulation of let-7b brings its levels to an intact iris (non-proliferative) state. That could also explain why proliferation of the ventral iris PECs is also seen by let-7b up-regulation.

Control of cell proliferation has been reported before for let-7 in breast cancer cell lines [14]. In fact similarly to our results over-expression of let-7 resulted in reduction of proliferation. Other miRNAs, such as miR-24, inhibit proliferation [15](see also below). Their up-regulation targets mostly cell cycle regulatory genes [15], [16]. Target genes for newt let-7 and miR-148 have not been identified due to lack of extensive sequence information. Because of that we decided to examine whether up-regulation of let-7b and miR-148 is able to regulate other miRNAs. Since target genes in other animals are known such regulation could allow us to make useful comparison and understand their function in regeneration.

### A miRNA network?

Because up-regulation of miR-148 or let-7b affected proliferation we collected cells after transfection, isolated RNA and probed a microarray containing all mouse miRNAs. Interestingly, we found that sets of miRNAs were regulated due to miRNA up-regulation (Table S1). More notable several of these miRNAs have been shown in other studies to be involved in the control of cell proliferation. Specifically, miR-30c [17], [18], miR-206 [19], [20], [21], miR-1 [20], [22], miR-10a [23], [24], [25], miR-146a [26], [27], [28], [29], miR-21 [30], [31], miR-130b [32] and miR-211 [33] (regulation by miR-148) have been shown to affect proliferation in various cell types and miR-203 to be involved in repression of stemness (Table 2). Likewise, miR-1 [20], [22], miR-200a [34], [35], let-7g [36], [37], [38], [39], [40], miR-9 [41] and miR-197 [42] (regulated by let-7b) have also been shown to affect proliferation (Table 3). Thus, we have identified another level of miRNA regulation, which indicated that miRNAs might act members of a network. This exciting idea needs of course to be verified in other systems as well.

**Table 2:** Cell proliferation related miRNAs, which were regulated by miR-148a.

miRNA	cells/tissues	Function	reference
miR-1	myoblasts; human-derived cardiomyocyte progenitor cells	muscle differntiation; NF-kappaB regulation	[20], [22]
miR-10a	human ovarian granulosa cells; megakaryocytic progenitors; chronic myeloid leukemia	reduces PCNA IR; be down-regulated during megakaryocytic differentiation; DR proliferation of CML	[23], [24], [25]
miR-21	liver regeneration; mesangial cells	UR in proliferative phase, targets Pellino-1, and inhibits NF-kappaB signaling; Over-expression of miR-21 inhibited proliferation	[30], [31]
miR-30c	hepatic organogenesis, connective tissue growth factor	required for hepatobiliary development, decreases CTGF levels, which was accompanied by decreased production of collagens	[17], [18]
miR-130b	Transformed human T-cell	NR of TP53INP1(cell growth factor)	[32]
miR-146a	cancer cells; megakaryocytosis; Myogenesis	negative regulation of NF-kappaB; decrease proliferation by NR of CXCR; NR of Numb	[26], [27], [28], [29]
miR-203	Mouse skin	tumor suppressor; repress stemness	[51], [52], [53], [54]
miR-206	rhabdomyosarcoma myoblasts; breast cancer cells,	suppresses c-Met expression; reduced cell proliferation and enhanced apoptosis; promotes muscle differentiation	[19], [20], [21]
miR-211	oral carcinoma cells	Enforced miR-211 increases the proliferation	[33]

**Table 3:** Cell proliferation related miRNAs, which were regulated by let-7b.

miRNA	cells/tissues	Function	reference
miR-1	myoblasts; human-derived cardiomyocyte progenitor cells	muscle differntiation; NF-kappaB regulation	[20], [22]
miR-9	human gastric adenocarcinoma;	inhibits NF-kappaB1	[41]
miR-197	follicular thyroid carcinoma cells	overexpression causes cell proliferation	[42]
miR-200a	nasopharyngeal carcinoma cell; brain tumor cells	inhibits cell growth, migration and invasion; inhibits translation and blocking Wnt/beta-catenin signaling	[34], [35]
let-7g	cencer cells; hepatocellular carcinoma Cells;	inhibits proliferation by down-regulation of c-Myc and Up-regulation of p16(INK4A)	[36], [37], [38], [39], [40]

### miRNAs, reprogramming, and dedifferentiation

An interesting connection between miRNAs and reprogramming of stem cells and of oocytes has been reported recently that might bear significance for the process of dedifferentiation during lens regeneration. Expression of let-7 in mammalian stem cells is evident before cells differentiate [43], [44]. In other words induction of let-7 prevents stemness and induces differentiation, while reduction up-regulates stemness factors and stemness. Likewise, during reprogramming of oocyte growth miRNA function is globally suppressed [45]. Among the suppressed miRNAs all members of the let-7 family are included. Interestingly, such a down-regulation of all let-7 members has also been seen during the dedifferentiation process of PECs as well as during repair of zebrafish retina [6], [46]. In addition, several pluripotency-maintaining factors, such as Sox-2, Klf4 and c-myc are expressed in the dorsal iris during dedifferentiation [47]. It has been suggested before that similar mechanisms could be involved in reprogramming of mammalian cells and their induction to iPSCs and reprogramming during regeneration in newts or zebrafish. The let-7 story is yet another interesting similarity between these two events.

### miRNAs and regeneration

Our previous study on the expression of miRNAs during lens and hair cell regeneration in newts was the first to indicate a possible involvement and regulation. The present study shows that, indeed, miRNAs can have a role in regeneration. Other recent reports support this as well. During zebrafish fin regeneration it has been shown that miR-203 regulates the Wnt signaling pathway transcription factor Lef1 [48]. Down-regulation of Lef1 by miR-203 blocks regeneration, while loss of miR-203 results in up-regulation of Lef1 and fin overgrowth. In another study it was found that depletion of miR-133 promotes fin regeneration and that this is FGF-dependent [49]. In a different study it was shown that miR-196 is involved in axolotl tail regeneration. Inhibition of miR-196 blocks regeneration by acting up-stream BMP4 and Pax-7 [50].

In this paper we describe the effects of two miRNAs on newt lens regeneration. We conclude that they differentially control cell proliferation in dorsal and ventral iris. Further, we identified that up-regulation of these two miRNAs results in regulation of other miRNAs. This is the first time that such regulation is reported and uncovers yet an unsuspected mode of miRNAs regulation as a network. Interestingly, many of the regulated miRNAs are associated with proliferation, which was the most prominent effect of miR-148 and let-7b. Our findings open new avenues in the study of miRNAs in cell proliferation, as is related to differentiation and regeneration.

## Supporting Information

Table S1 miRNA microarrays. The files present raw data on miRNA microarrays. Ventral PECs were up-regulated with let-7b or miR-148 by transfection and isolated RNA was used to probe microarrays. Control samples were PECs transfected with scrambled miRNAs (see Methods).
(0.04 MB XLS)
